# Structural basis for the inhibitory effects of a novel reversible covalent ligand on PPARγ phosphorylation

**DOI:** 10.1038/s41598-019-47672-w

**Published:** 2019-08-01

**Authors:** Jun Young Jang, Hyunsoo Kim, Hyun-Jung Kim, Se Won Suh, Seung Bum Park, Byung Woo Han

**Affiliations:** 1 Research Institute of Pharmaceutical Sciences, College of Pharmacy, Seoul National University, Seoul, 08826 Republic of Korea; 2 Department of Chemistry, College of Natural Sciences, Seoul National University, Seoul, 08826 Republic of Korea; 3 College of Pharmacy, Chung-Ang University, Seoul, 06974 Republic of Korea

**Keywords:** Drug discovery, X-ray crystallography, Type 2 diabetes

## Abstract

Peroxisome proliferator-activated receptor γ (PPARγ) is a major therapeutic target for the treatment of type 2 diabetes. However, the use of PPARγ-targeted drugs, such as rosiglitazone and pioglitazone, is limited owing to serious side effects caused by classical agonism. Using a rational drug discovery approach, we recently developed SB1495, a novel reversible covalent inhibitor of the cyclin-dependent kinase 5 (Cdk5)-mediated phosphorylation of PPARγ at Ser245, a key factor in the insulin-sensitizing effect of PPARγ-targeted drugs. In this study, we report the crystal structures of PPARγ in complex with SB1495 and its enantiomeric analogue SB1494, which rarely exhibits inhibitory activity, to visualize the mechanistic basis for their distinct activities. SB1495 occupies the Arm3 region near the Ω loop of the PPARγ ligand-binding domain, whereas its enantiomeric analogue SB1494 binds to the Arm2 region. In addition, the piperazine moiety of SB1495 directly pushes the helix H2′, resulting in the stabilization of the Ω loop just behind the helix H2′. Our results may contribute to the development of a new generation of antidiabetic drugs that selectively block PPARγ phosphorylation without classical agonism.

## Introduction

Peroxisome proliferator-activated receptors (PPARs) are ligand-activated transcription factors belonging to the thyroid hormone receptor-like nuclear receptor subfamily 1^1^. PPARs form heterodimers with retinoid X receptors (RXRs), recruit coactivators, and subsequently bind to specific sequences on target genes, termed peroxisome proliferator response elements, thereby regulating transcription^2^. There are three PPAR subtypes, PPARα, PPARγ, and PPARδ/β^3^. PPARγ is mainly expressed in the adipose tissue, colon, and macrophages and plays important roles in adipocyte differentiation, lipid metabolism, glucose homeostasis, insulin sensitization, and inflammatory action^4,5^. Thus, PPARγ is one of the most effective pharmacological targets for type 2 diabetes mellitus as well as other metabolic diseases, such as obesity and atherosclerosis^6^.

The ligand-binding pocket (LBP) of PPARγ is a Y-shaped pocket with a volume of 1300–1440 Å^3^, which is much larger than those of other nuclear receptors^7,8^. The left arm (Arm1) of the Y-shaped pocket is composed of a mix of hydrophobic residues and polar residues and is covered by the activation function-2 (AF-2) helix (i.e., helix H12). The right arm (Arm2) of the Y is composed of hydrophobic residues and is parallel to helix H3. The third arm (Arm3) of the Y-shaped pocket is composed of hydrophobic residues and is surrounded by helix H2′, a Ω loop, and a four-stranded β-sheet^9,10^.

PPARγ modulates transcriptional activity in response to ligand binding by the recruitment of coactivators^7^. Thiazolidinediones (TZDs), such as rosiglitazone, pioglitazone, and lobeglitazone, are PPARγ agonists that interact with Tyr473 on the AF-2 helix^11^. However, full PPARγ agonists are used with caution owing to their known adverse effects, including weight gain, increased adipogenesis, renal fluid retention, and plasma volume expansion^12,13^.

The antidiabetic effects of PPARγ agonists have been well understood. However, the mechanism of the side effects caused by PPARγ agonists is poorly understood, and thus insulin-sensitizing agents targeting PPARγ with minimal side effects still need to be developed. Recently, inhibitors that selectively block the cyclin-dependent kinase 5 (Cdk5)-mediated phosphorylation of PPARγ at Ser245 (in PPARγ1; Ser273 in PPARγ2) without classical transcriptional activation have been developed^14,15^. Studies have also focused on covalent synthetic ligands for PPARγ to enhance the binding affinity and biological activity, since some endogenous unsaturated fatty acids, oxidized fatty acids, and nitrated fatty acids covalently bind to Cys285 in the PPARγ ligand-binding domain (LBD) via Michael addition by a thiol moiety and modulate PPARγ activity^8,16^. As alternative antidiabetic agents, selective inhibitors of the phosphorylation of PPARγ at Ser245 have been developed by introducing an irreversible covalent bond between ligands and Cys285 in PPARγ LBP^17^. However, irreversible covalent drugs could elicit “off-target” effects from the irreversible modification of unexpected target nucleophiles that may cause toxicities^18^.

Using a rational drug discovery approach, we recently developed a novel reversible covalent inhibitor of the Cdk5-mediated phosphorylation of PPARγ at Ser245, i.e., SB1495, which could avoid adverse effects caused by the irreversible covalent modification of off-target nucleophiles in biological systems^19^. While most other rationally designed covalent ligands of PPARγ contain the nitroarene moiety that links the ligands to PPARγ irreversibly^20–22^, SB1495 is the first compound that contains the cyanoacrylamide moiety, which allows the reversible covalent bond formation to PPARγ. To elucidate the structural basis for the inhibitory activity of SB1495 and to provide essential structural information for the improvement of reversible covalent inhibitors as antidiabetic agents, we determined the crystal structures of PPARγ LBD in complex with two enantiomeric reversible covalent ligands, SB1495 and SB1494. Unexpectedly, these enantiomeric ligands bind to PPARγ LBD in distinct manners. SB1495 occupies the Arm3 region near the Ω loop, whereas its enantiomeric analogue SB1494 binds to the Arm2 region. In addition, the piperazine moiety of SB1495 directly pushes helix H2′, resulting in a significant change in helix H2′ and a stable conformation of the Ω loop located just behind helix H2′. These results may lead to the development of new antidiabetic drugs that block Cdk5-mediated PPARγ phosphorylation at Ser245.

## Results

### Discovery and bioactivity of reversible covalent ligands, SB1495 and SB1494, of PPARγ phosphorylation

**Figure 1:**
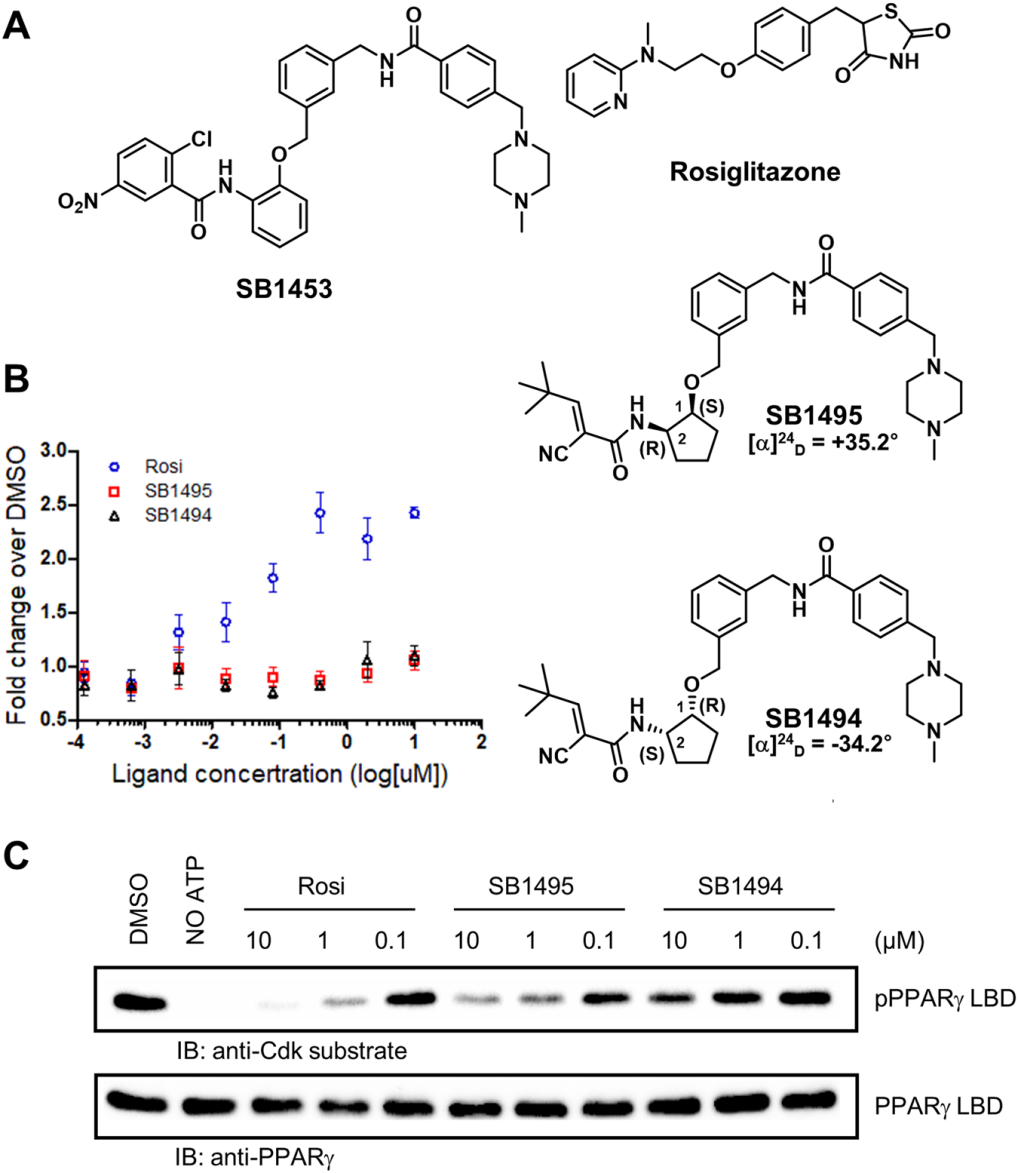
Structures and bioactivity of reversible covalent ligands SB1495 and SB1494. (**A**) Chemical structures of SB1453, rosiglitazone, SB1495, and SB1494. Stereochemistry of SB1495 (1S, 2R) and SB1494 (1R, 2S) is labeled in the proximity of the chiral carbons. The optical rotation values that were measured on a JASCO P-1030 polarimeter at the sodium D line are presented below SB1495 and SB1494 labels. (**B**) Transcriptional activity of a PPARγ-derived reporter gene in HEK-293T cells expressing full-length PPARγ after 20 h of treatment with rosiglitazone, SB1495, and SB1494. (**C**) *In vitro* Cdk5-mediated PPARγ phosphorylation assay with rosiglitazone, SB1495, and SB1494 in a dose-dependent manner.

As alternative antidiabetic agent, SB1453, a selective inhibitor of the phosphorylation of PPARγ at Ser245 has been developed by introducing an electrophile that forms an irreversible covalent bond with Cys285 in helix H3 of PPARγ LBD^17^ (Fig. 1A). However, the irreversible covalent inhibitor SB1453 containing a nitroaryl moiety has some weaknesses for biological applications, such as side effects related to its partial agonism and cytotoxicity at high concentrations^17^. Therefore, we recently developed a series of reversible covalent PPARγ ligands containing a cyanoacrylamide moiety^19^. Among them, SB1495 that contains a cyanoacrylamide moiety connected to (1S, 2R)-2-aminocyclopentan-1-ol exhibited the most effective inhibitory activity against PPARγ (Fig. 1A). Notably, 2-aminocyclopentan-1-ol has four stereoisomers (SB1494–1497) (Supplementary Fig. S1A), and only (1S, 2R) conformer in SB1495 has potent inhibition activity towards PPARγ phosphorylation^19^. We performed a MALDI-TOF analysis to determine whether four stereoisomers SB1494–1497 bind to PPARγ LBD. As shown in Supplementary Fig. S1, we observed that SB1495 and SB1494 show selective binding to PPARγ LBD; they competitively blocked the covalent binding of SB1453 to PPARγ LBD by occupying its binding pocket. SB1496 and SB1497 were not able to interrupt the covalent complexation of SB1453 with PPARγ LBD. We also observed that neither SB1495 nor SB1494 induce agonistic activity (Fig. 1B). Therefore, we concluded that both SB1495 and SB1494 are antagonistic ligands of PPARγ. However, only SB1495 inhibited the Cdk5-mediated phosphorylation of PPARγ *in vitro* (Fig. 1C). Thus, we evaluated the mechanistic difference between SB1495 and SB1494 at the molecular level and their structural properties by determining the co-crystal structures of PPARγ LBD in complex with SB1495 and with SB1494.

### Overall structures of SB1495-bound and SB1494-bound PPARγ LBD

**Figure 2:**
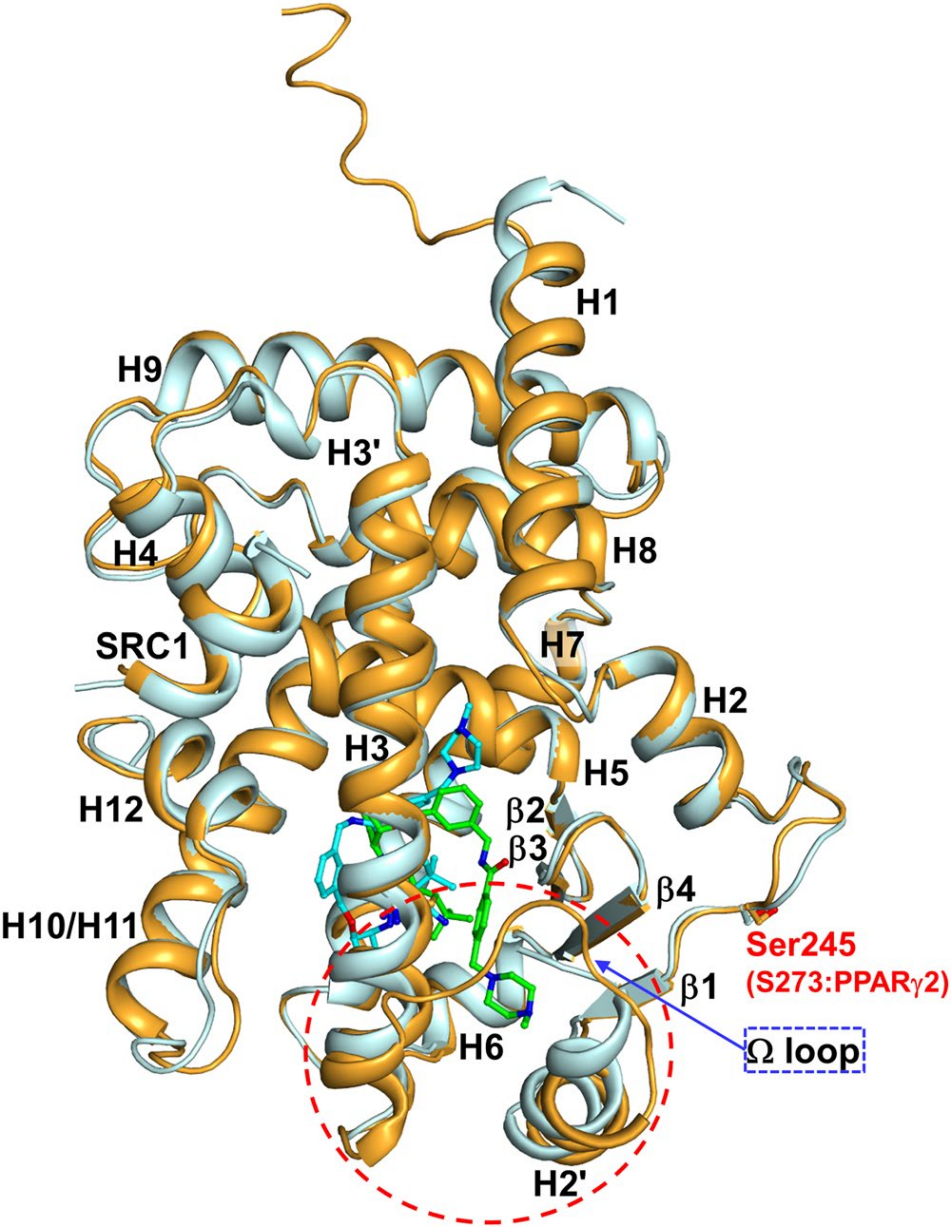
Overall structures of SB1495-bound and SB1494-bound PPARγ LBD. Superposition of PPARγ LBD structures of SB1495-bound (bright orange) and SB1494-bound (pale cyan) models. Structures of SB1495-bound and SB1494-bound PPARγ LBD contain the SRC-1 peptide colored same with bound PPARγ LBD structures. SB1495 and SB1494 are displayed by green and cyan ball-and-stick models, respectively. The Cdk5-mediated phosphorylation site Ser245 is displayed by a ball-and-stick model. Helix H2′ and the Ω loop which exhibited large conformational changes are depicted by a red-dashed circle.

SB1495 and SB1494 are enantiomers of the 2-aminocyclopentan-1-ol moiety (Fig. 1A). The optical rotation values ([*α*]^24^_D_) of SB1495 and SB1494 were +35.2 and −34.2, respectively, which confirm their enantiomeric relationship. To gain insights into the binding modes of these enantiomeric reversible covalent ligands, we determined the crystal structures of PPARγ LBD in complex with SB1495 and with SB1494 in the presence of a peptide derived from human steroid receptor coactivator-1 (SRC-1) at 2.85 Å and 2.15 Å resolution, respectively. Crystals of SB1495-bound PPARγ LBD belonged to the *orthorhombic* space group *P*2_1_2_1_2_1_ with the unit cell parameters a = 62.4 Å, b = 62.5 Å, c = 162.2 Å and contained two monomers in an asymmetric unit, whereas crystals of SB1494-bound PPARγ LBD belonged to the *orthorhombic* space group *P*2_1_2_1_2 with the unit cell parameters a = 131.2 Å, b = 53.3 Å, c = 53.8 Å and contained one monomer in an asymmetric unit (Supplementary Fig. S2). Two SB1495-bound PPARγ LBD monomers in the asymmetric unit were highly similar to each other, with root-mean-square deviation (RMSD) values of 0.20 Å for 197–477 C_α_ atom pairs. The overall structures of PPARγ LBD in complex with SB1495 and with SB1494 were similar, with RMSD of 1.64 Å for 263 C_α_ atoms. The structures adopted a canonical fold of general nuclear receptors including a bundle of 13 α-helices and a four-stranded mixed β-sheet (β1↓–β4↓–β3↑–β2↓) (Fig. 2). The C-terminal helix H12 in the active conformation is folded toward helices H3 and H11 and covers the LBP of PPARγ. The canonical helical LxxLL motif from the SRC-1 peptide makes contacts with a hydrophobic groove consisting of residues from helices H3, H4, H5, and H12 of PPARγ LBD (Fig. 2). The Cdk5-mediated phosphorylation site Ser245 of SB1495-bound and SB1494-bound PPARγ LBD did not exhibit noticeable conformational differences with RMSD of 0.57 Å for Ser245 C_α_ atom and the same rotamer orientation. However, when SB1495 binds to PPARγ LDB, it induces conformational changes in helix H2′ and the Ω loop compared with the SB1494-bound PPARγ LBD structure (Fig. 2).

We tried to determine the SB1495-bound and SB1494-bound structures by a soaking method from the same crystallization condition to exclude any possibility that crystal packing affects structural changes. Unfortunately, we could not observe reasonable electron densities of ligands from the soaking method. Alternatively, we determined the structure of ligand-free PPARγ LBD from the same crystallization condition for the structure of SB1495-bound PPARγ LBD by co-crystallization with the coactivator SRC-1 peptide (60% *v/v* Tacsimate, pH 7.0). However, the crystals of ligand-free PPARγ LBD belonged to the space group *P*2_1_2_1_2 with the unit cell parameters nearly identical to the crystals of SB1494-bound PPARγ LBD (Supplementary Table S1). In addition, the previously reported structure of R35-bound PPARγ LBD were determined from a similar crystallization condition (57.5% *v/v* Tacsimate, pH 7.0) to that of SB1495-bound PPARγ LBD^10^, and the crystals of R35-bound PPARγ LBD also belonged to the same space group *P*2_1_2_1_2 with the crystals of ligand-free PPARγ LBD (Supplementary Table S1). Since the crystals of ligand-free, SB1494-bound, and R35-bound PPARγ LBD belonged to the same space group with similar unit cell parameters, the SB1495 binding to PPARγ LBD would induce the structural changes in helix H2′ and the Ω loop, resulting in a different crystal packing.

### Covalent binding mode in the SB1495- and SB1494-bound PPARγ structures

**Figure 3:**
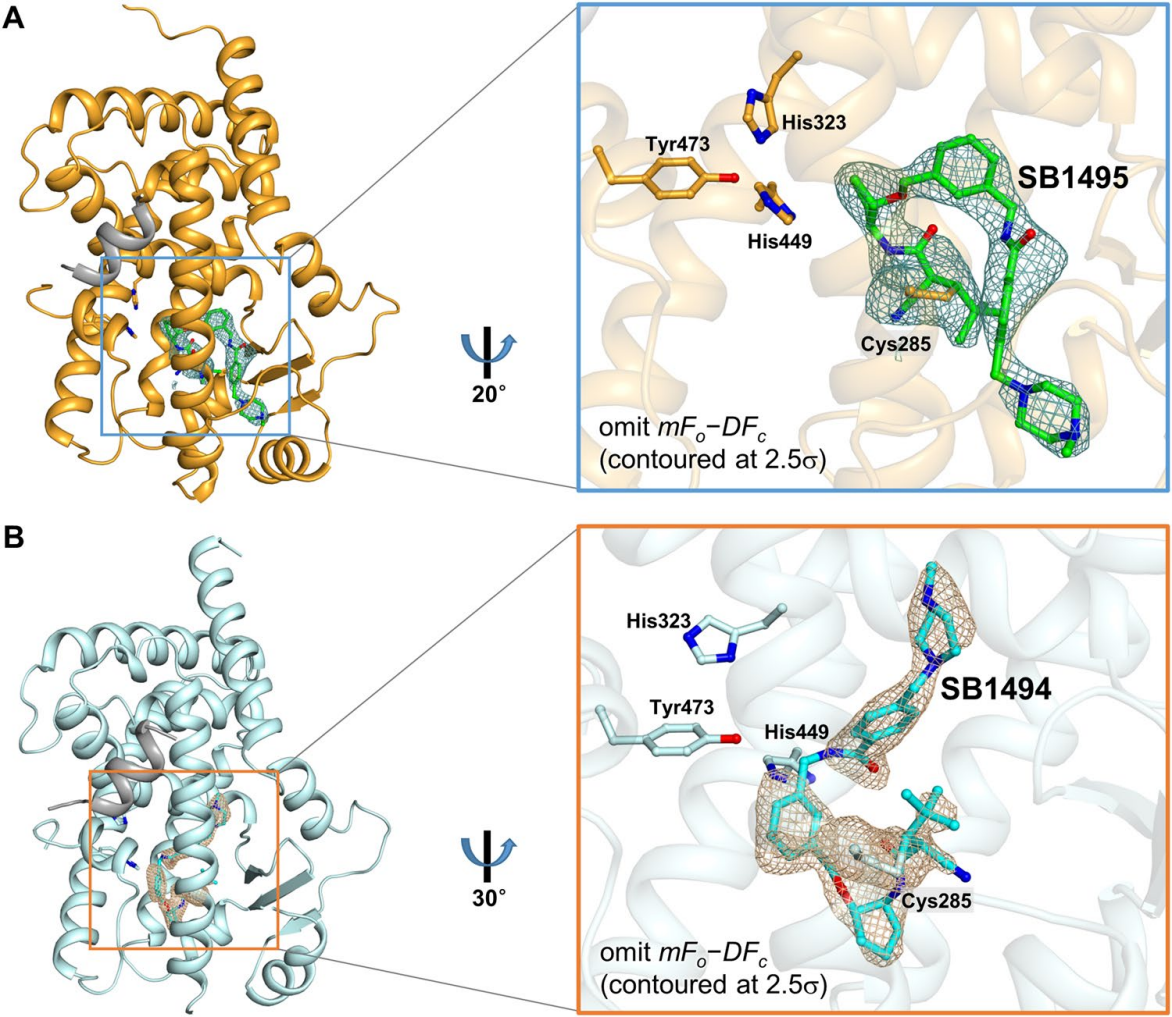
Covalent binding modes in the SB1495-bound and SB1494-bound PPARγ LBD structures. (**A**) Ribbon model of SB1495-bound PPARγ LBD (bright orange) in complex with the SRC-1 peptide (grey). SB1495, displayed as a green ball-and-stick model, occupies the LBP of PPARγ. The *mFo-DFc* electron density omit map contoured at 2.5σ for SB1495 is represented as a light teal-colored mesh. The right panel shows a magnified image of interactions between PPARγ Cys285 and SB1495. (**B**) Ribbon model of SB1494-bound PPARγ LBD (pale cyan) in complex with the SRC-1 peptide (grey). SB1494, displayed as a cyan ball-and-stick model, is represented in the same way as SB1495 (**A**).

SB1495 and SB1494 have a cyanoacrylamide moiety capable of forming a reversible covalent bond with a thiol moiety of Cys285 in PPARγ LBD under physiological conditions^23^ (Fig. 1A). In the structures of SB1495- and SB1494-bound PPARγ LBD, the omit maps calculated from the refined models clearly revealed extra electron densities that could be modeled as SB1495 and SB1494, respectively. The structures confirmed that both SB1495 and SB1494 form covalent bonds as a predicted mechanism, with strong electron densities connecting Cys285 to the electrophilic β-carbon of the cyanoacrylamide moiety (Fig. 3 and Supplementary Fig. S3). The interactions between SB1495 and PPARγ LBD involve one hydrogen bond and hydrophobic effects (Supplementary Fig. S4A). SB1495 forms a hydrogen bond with the backbone nitrogen of Ser342 on strand β3 with a distance of 2.7 Å. Hydrophobic effects between SB1495 and PPARγ LBD involve residues Glu259, His266, Thr268, Ile281, Phe282, Gly284, Arg288, Ser289, Tyr327, Leu330, Leu333, Leu340, Ile341, Met348, Leu353, and Met364. The interactions between SB1494 and PPARγ LBD involve only hydrophobic effects (Supplementary Fig. S4B). As shown in Fig. 3, we found that the moiety from cyanoacrylamide to cyclopentane of SB1495 extends from the pivotal Cys285 towards the C-terminus of helix H3 and then turns to the N-terminus of helix H3. In contrast, the equivalent moiety of SB1494 extends from the pivotal Cys285 towards the N-terminus of helix H3 and then turns to the C-terminus of helix H3. These enantiomeric ligands SB1495 and SB1494 did not form a hydrogen bond network with His323, His449, and Tyr473, which are generally presented in the PPARγ full agonist-bound structure. Additionally, in the SB1494-bound structure, SB1494 bound closely to the canonical pocket formed by His323, His449, and Tyr473, resulting in a conformational change of His323 (Fig. 3). In order to compare the covalent binding modes of SB1495 and SB1494 with other covalent PPARγ ligands, we superimposed SB1495- and SB1494-bound structures with a total of 22 covalent ligands for PPARγ LBD deposited in Protein Data Bank (PDB) so far^8,17,24–29^. Compared with other covalent ligands, we observed that the piperazine moiety of SB1495 is specifically protruded towards helix H2′ in PPARγ LBP (Supplementary Fig. S5).

### SB1495 binding induced structural changes of helix H2′ connected to the Ω loop

**Figure 4:**
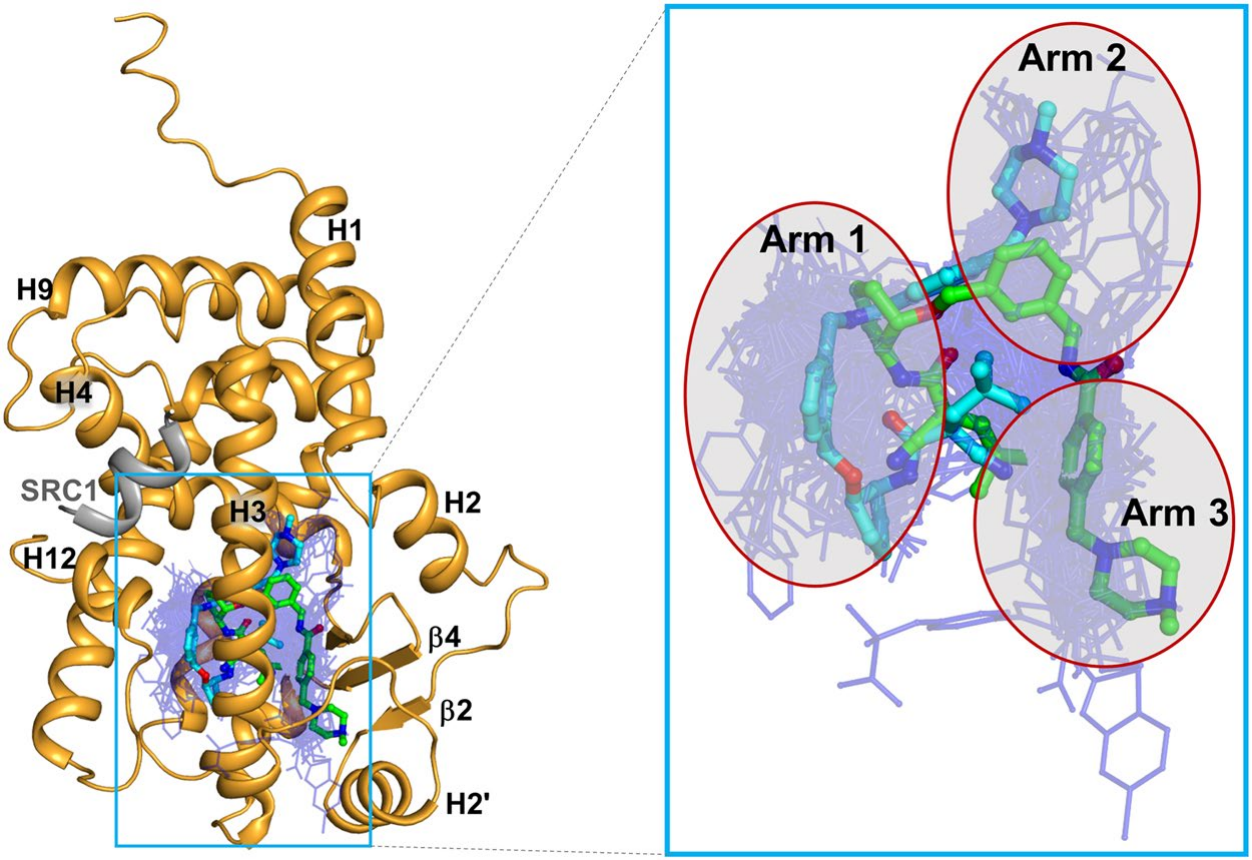
Superposition of SB1495 and SB1494 with other PPARγ ligands from known complex structures. A total of 138 ligand-bound PPARγ LBD structures in PDB were superposed onto the SB1495-bound structure (bright orange). Green and cyan ball-and-stick models indicate SB1495 and SB1494, respectively, and blue lines represent the other ligands. Right panel shows a magnified image of PPARγ LBP. Characteristic ligand-binding regions Arm1, Arm2, and Arm3 in PPARγ LBP are highlighted in red circles.

**Figure 5:**
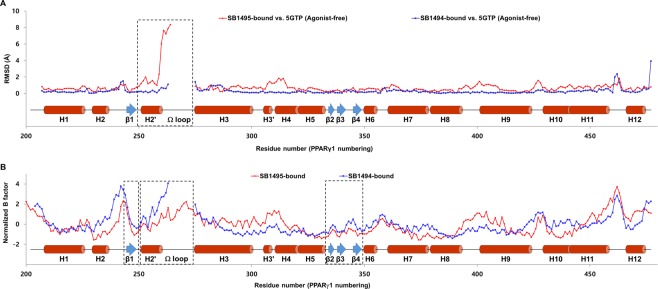
Comparison of the SB1495-bound and SB1494-bound structures with respect to conformation and stability. (**A**) Comparison of the C_α_ RMSD values for the SB1495- and SB1494-bound structures against the agonist-free PPARγ LBD structure (PDB ID: 5GTP). Red and blue lines represent the RMSD values for the SB1495-bound and SB1494-bound PPARγ LBD structures, respectively. Secondary structural elements are represented along the residue numbers. Helix H2′ and the Ω loop which exhibited large conformational changes are marked by a black-dashed box. (**B**) Comparison of the normalized B-factors for the SB1495- and SB1494-bound structures. The normalized B-factors for the SB1495- and SB1494-bound PPARγ LBD structures are represented in red and blue lines, respectively. Helix H2′, the Ω loop, and the four-stranded β-sheet which exhibited enhanced thermal stabilities in the SB1495-bound structure are marked by black-dashed boxes.

**Figure 6:**
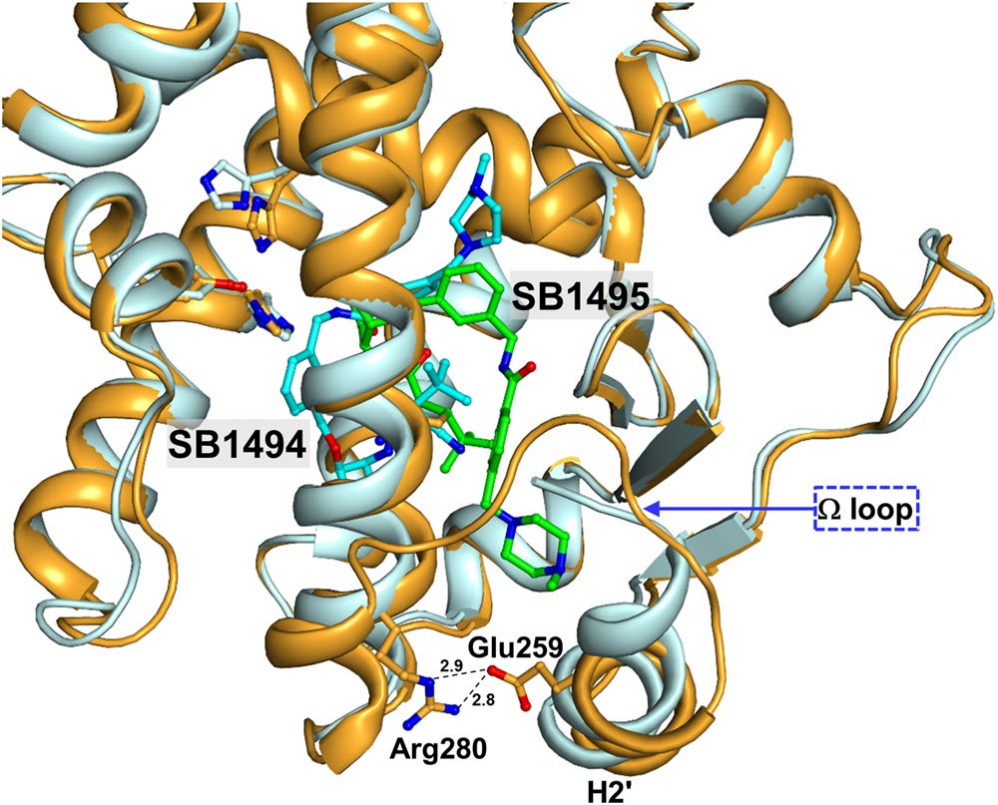
The magnified image of interactions between SB1495 and helix H2′ of PPARγ. SB1495 and SB1494 are represented by green and cyan ball-and-stick models, respectively. Glu259 and Arg280 that form hydrogen bonds in the SB1495-bound structure are shown as bright orange ball-and-stick models. Oxygen and nitrogen atoms are colored in red and blue, respectively. Dashed lines depict hydrogen bonds and the corresponding hydrogen bond distances (Å) are labeled.

We superimposed the structures of many different ligands taken from 138 PPARγ LBD structures onto our SB1495- and SB1494-bound PPARγ LBD structures (Fig. 4). As shown in Fig. 4, PPARγ ligands superposed in PPARγ LBP form Arm1, Arm2, and Arm3 regions. SB1495 occupied the Arm3 region of the LBP near the alternate binding site of PPARγ LBD. However, SB1494, an enantiomeric analogue of SB1495, occupied the Arm2 region of PPARγ LBP. The Ω loop between helices H2′ and H3 is very flexible based on PPARγ LBD structures solved to date and the Ω loop of SB1494-bound PPARγ LBD structure could not be modeled owing to the low electron densities. To our surprise, the Ω loop of SB1495-bound PPARγ LBD structure was reasonably well-defined by the electron density and residues 271–274 (Gln-Glu-Gln-Ser) in the Ω loop formed a helix. When we compared the C_α_ RMSD values for SB1495-bound vs. agonist-free PPARγ LBD (PDB ID: 5GTP) and SB1494-bound vs. agonist-free PPARγ LBD structures, we observed a unique conformational change (Fig. 5A). The piperazine moiety of SB1495 directly pushed helix H2′, resulting in a significant shift in helix H2′ compared with the SB1494-bound structure. In addition, a hydrogen bond formed between Glu259 on helix H2′ and Arg280 on helix H3 due to the shift in helix H2′ (Fig. 6). Next, we analyzed B factors to obtain insight into protein dynamics after ligand binding. The comparison of normalized B factors revealed that the binding of SB1495 in the LBD induces a stable conformation of helix H2′, the Ω loop, and the four-stranded β-sheet compared to the binding of SB1494 (Fig. 5B).

## Discussion

Full PPARγ agonists, such as TZD drugs, are potent insulin sensitizers for the treatment of type 2 diabetes mellitus, but their side effects have been problematic, including fluid retention, edema, bone loss, and weight gain^30^. There have been many attempts to produce safer versions of PPARγ-specific drugs that preserve insulin-sensitizing effects without adverse effects. It is extremely important to understand the molecular mechanism underlying PPARγ regulation for the development of novel and safe PPARγ-based antidiabetic agents. The phosphorylation of PPARγ at Ser245 by Cdk5 alters the expression of specific genes, such as adiponectin, which is associated with insulin sensitivity, unlike the general transcriptional agonism of PPARγ^14^. PPARγ has a large LBP and numerous PPARγ ligands can be accommodated in a variety of binding modes to regulate PPARγ activities. In addition, one or more of the ligands can be accommodated in PPARγ LBP and the regulation of PPARγ activity by bound ligands is a fairly complex process^8,21^. In this respect, the development of synthetic ligands that form covalent linkages with Cys285 located in the center of PPARγ LBP will be very useful for the selective modulation of PPARγ.

The reversible covalent PPARγ ligands, SB1495 and SB1494, lacked agonistic activity on PPARγ-mediated transcription (Fig. 1B). Since SB1495 inhibited the phosphorylation of PPARγ at Ser245 and its enantiomeric analogue SB1494 rarely did (Fig. 1C), we compared the crystal structures of PPARγ in complex with SB1495 and with SB1494. Both SB1495 and SB1494 bound to PPARγ LBD by forming covalent bonds with Cys285 on helix H3 and did not show any interaction with helix H12, which is important for the canonical transcriptional activity of PPARγ (Fig. 3). Given these results, the selective inhibition of PPARγ phosphorylation by reversible covalent synthetic ligands could be a powerful strategy for the development of a novel antidiabetic agents targeting PPARγ.

Even though SB1495 and SB1494 are enantiomers in the 2-aminocyclopentan-1-ol moiety, their binding modes to PPARγ LBD were quite different. SB1495 and SB1494 bound to the Arm3 region and to the Arm2 region, respectively (Fig. 4). These observations are similar to those for other structural isomers SB1405 and SB1406, which differ significantly with respect to biological activities in PPARγ phosphorylation^17^. This difference in binding modes could affect the phosphorylation of PPARγ at Ser245. Ligand binding at the specific binding site in the Arm3 region between helix H3 and strands β3–β4 appears to be closely related to the inhibition of PPARγ phosphorylation at Ser245, consistent with the difference between SB1405 and SB1406 reported previously^17^. Protein kinases are substrate-specific and kinase substrates are known to have contiguous consensus motifs that are linear sequences adjacent to the phosphorylation site^31,32^. However, it has been also reported that noncontiguous consensus motifs structurally formed in the three-dimensional structure of a substrate phosphorylation site can be recognized by protein kinases^33^. This suggests that conformational changes around the Cdk5-mediated phosphorylation site (Ser245) of PPARγ may affect the activity of Cdk5. In fact, the Ω loop and the β-sheet site (residues Asn335-Asp337, Thr349, and Glu351) of PPARγ are structurally close to the Ser245 phosphorylation site and they were predicted to affect the binding of Cdk5 to PPARγ according to the proposed molecular recognition model for the PPARγ-Cdk5/p25 complex using protein-protein docking and molecular dynamics simulations^34^. The binding of SB1495 to PPARγ LBD induces a stable conformation of helix H2′, the Ω loop, and the four-stranded β-sheet (Fig. 5B), which may interfere with the binding of PPARγ to Cdk5 and thus affect the Cdk5-mediated phosphorylation of PPARγ (Fig. 1C).

Morikawa and colleagues intensively analyzed structural aspects of PPARγ transcriptional activation when endogenous fatty acids form a covalent bond with PPARγ. Conformational changes in the Ω loop induced by covalent ligands affected the modulation of PPARγ transcriptional activity^35^. In addition, PPARγ structures in complex with various endogenous and synthetic covalent ligands have been studied for the regulation of PPARγ function and development of antidiabetic drugs^8,17,24–29^. So far, there are 22 covalent PPARγ ligands deposited in PDB. When we compared all the binding modes of 22 known covalent ligands from PPARγ-complexed structures, we found that the characteristic piperazine moiety of SB1495 directly pushed helix H2′, resulting in a noticeable shift in helix H2′, unlike other known covalent ligands from PPARγ-complexed structures (Fig. 6 and Supplementary Fig. S5). In addition, the structure of the Ω loop was well defined and adopted a four-amino acid-long helix at the C-terminus of the Ω loop in the SB1495-bound PPARγ LBD structure compared with the SB1494-bound structure (Fig. 6). Thus, detailed structural analyses using covalent ligands, such as SB1495, will extend our knowledge about the regulation of complex PPARγ functions.

PPARγ functions are also regulated by post-translational modifications, including acetylation, sumoylation, and ubiquitination, as well as phosphorylation^30^. PPARγ acetylation on Lys240 and Lys265 was regulated by the NAD^+^-dependent deacetylase SirT1 and it influences the ‘browning’ of white adipose tissue. The deacetylation of PPARγ tilts the balance from energy storage to energy expenditure and promotes insulin sensitivity^36^. Lys265 is located in the Ω loop, and in-depth studies of conformational changes of the Ω loop are likely to be important for determining the selective biological functions of PPARγ. Taken all together, our structures and biological results may contribute to the development of a new generation of antidiabetic drugs that selectively block PPARγ phosphorylation without classical agonism.

## Methods

### Chemistry

SB1494, SB1495, SB1496, and SB1497 were synthesized as previously reported^19^.

### Cell culture

HEK-293T human embryonic kidney cells were purchased from the American Type Culture Collection (Manassas, VA, USA). HEK-293T cells were cultured in Dulbecco’s modified Eagle’s medium containing 10% fetal bovine serum and 1% *v/v* antibiotic-antimycotic solution.

### *In vitro* kinase assay

An *in vitro* kinase assay of PPARγ was performed as previously reported^17^. In brief, 0.5 μg of the recombinant PPARγ LBD was incubated with an active form of Cdk5/p35 (Eurofins Scientific, Dundee, United Kingdom) in assay buffer (25 mM Tris-HCl, pH 7.5, 2 mM DTT, 10 mM MgCl_2_, 0.1 mM Na_3_VO_4_ and 5 mM β-glycerophosphate) containing 25 μM ATP at 30 °C. SB1495 or SB1494 were pre-treated with PPARγ LBD at 30 °C, and the assay was conducted. Proteins were separated by SDS-PAGE, and the phospho-serine in a consensus motif was identified using an anti-Cdk substrate antibody, Phospho-CDK Substrate Motif [(K/H)pSP] MultiMab Rabbit mAb (Cell Signaling Technology, Danvers, MA, USA).

### Cell-based luciferase reporter gene assay

For transfection assay, HEK-293T cells were seeded in 96-well plates at a density of 7 × 10^3^ cells per well. After 24 h, the cells were transiently cotransfected with a pDR-1 luciferase reporter plasmid, PPARγ, RXRα, and pRL-renillin using the calcium phosphate method. Transfected cells were treated with various concentrations of rosiglitazone, SB1495, or SB1494 for 20 h. The cells were harvested with a cell lysis buffer, and the Dual Luciferase Kit (Promega, Madison, WI, USA) were used to measure the luciferase activity with a Bio-Tek microplate reader ELx800^TM^ (Bio-Tek Instruments Inc., Winooski, VT, USA). The luciferase activity was normalized by *Renilla* units. Fold change values of treated cells over DMSO-treated control cells were plotted in triplicate.

### Protein expression and purification

The protein expression, purification, and various crystallization of human PPARγ LBD were mainly implemented as previously reported^10,11^. In brief, the PPARγ LBD expression vector was transformed into the Rosetta 2(DE3) *Escherichia coli* strain. The transformed cells were grown to mid-log phase in Luria-Bertani medium at 37 °C and induced with 0.8 mM isopropyl β-d-thiogalactopyranoside at 18 °C. The cells were resuspended in a lysis buffer (150 mM NaCl, 10% *v/v* glycerol, 0.1 mM tris(2-carboxyethyl) phosphine hydrochloride, and 20 mM Tris-HCl, pH 8.5) containing 1 mM phenylmethylsulfonyl fluoride and 5 mM imidazole and lysed by sonication. The crude lysate was pelleted by centrifugation at 14,000 rpm for 50 min and the supernatant was loaded onto to a 5-mL HiTrap Chelating HP affinity chromatography column (GE Healthcare, Chicago, IL, USA). After washing with the lysis buffer, the recombinant PPARγ LBD protein was eluted with a gradient of 50–100 mM imidazole. The eluted protein was loaded onto a HiPrep 26/10 desalting column (GE Healthcare) and eluted with the lysis buffer. After cleavage with thrombin (Merck Millipore), the PPARγ LBD protein was further purified by gel filtration on a HiLoad 16/600 Superdex 200 pg chromatography column (GE Healthcare). Finally, the purified PPARγ LBD was concentrated to 15.6 mg/mL for crystallization.

### Crystallization

SB1495 or SB1494 were co-crystallized with PPARγ LBD and the coactivator SRC-1 peptide (ERHKILHRLLQEGSPS) by excessively adding seven times more than the PPARγ LBD concentration. After overnight incubation followed by crystallization, crystals of SB1495-bound and ligand-free PPARγ LBD were grown with a crystallization solution of 60% *v/v* Tacsimate, pH 7.0. Crystals of SB1494-bound PPARγ LBD were grown with a crystallization solution of 2.2 M sodium malonate, pH 7.0.

### X-ray data collection

X-ray data collection for SB1495-bound and SB1494-bound PPARγ LBD was implemented at the synchrotron beamline BL-7A of the Pohang Light Source (Pohang, Korea) at 100 K. X-ray data for ligand-free PPARγ LBD were collected at the synchrotron beamline BL-11C of the Pohang Light Source. Collected data were indexed, integrated, and scaled using *HKL2000*^37^. The space groups for crystals of SB1495-bound, SB1494-bound, and ligand-free PPARγ LBD were *P2*_1_2_1_2_1_, *P2*_1_2_1_2, and *P2*_1_2_1_2, respectively. Supplementary Table S2 summarizes the data collection statistics.

### Structure determination and refinement

Structures of SB-1495-bound, SB1494-bound, and ligand-free PPARγ LBD were determined using the molecular replacement method by *MolRep*^38^ with the agonist-free structure of PPARγ LBD (PDB ID: 5GTO) as the probe^10^. Subsequent manual model building was carried out with *COOT*^39^. The models were further refined in *REFMAC5*^40^, including bulk solvent correction. Five percent of the observed data were randomly excluded for the free *R* factor calculation^41^. The quality of the final models was evaluated using *MolProbity*^42^. Supplementary Table S2 summarizes the refinement statistics.

### Accession codes

The atomic coordinates of the final models and experimental structure factors have been deposited in the Protein Data Bank (PDB ID 6IJR, 6IJS, and 6JQ7 for the SB1495-bound, SB1494-bound, and ligand-free PPARγ LBD structures, respectively).

## Supplementary information

Supplementary information
