# Publisher Correction: Structural basis for the inhibitory effects of a novel reversible covalent ligand on PPARγ phosphorylation

**DOI:** 10.1038/s41598-019-50831-8

**Published:** 2019-10-29

**Authors:** Jun Young Jang, Hyunsoo Kim, Hyun-Jung Kim, Se Won Suh, Seung Bum Park, Byung Woo Han

**Affiliations:** 1 Research Institute of Pharmaceutical Sciences, College of Pharmacy, Seoul National University, Seoul, 08826 Republic of Korea; 2 Department of Chemistry, College of Natural Sciences, Seoul National University, Seoul, 08826 Republic of Korea; 3 College of Pharmacy, Chung-Ang University, Seoul, 06974 Republic of Korea

Correction to: *Scientific Reports* 10.1038/s41598-019-47672-w, published online 01 August 2019

In the original version of this Article, Jun Young Jang and Hyunsoo Kim were omitted as equally contributing authors.

In addition, this Article contained a typographical error in the Results section, under subheading ‘Covalent binding mode in the SB1495- and SB1494-bound PPARγ structures’.

“In order to compare the covalent binding modes of SB1495 and SB1494 with other covalent PPARγγ ligands, we superimposed SB1495- and SB1494-bound structures with a total of 22 covalent ligands for PPARγ LBD deposited in Protein Data Bank (PDB) so far^8,17,24–29^.”

now reads:

“In order to compare the covalent binding modes of SB1495 and SB1494 with other covalent PPARγ ligands, we superimposed SB1495- and SB1494-bound structures with a total of 22 covalent ligands for PPARγ LBD deposited in Protein Data Bank (PDB) so far^8,17,24–29^.”

These errors have now been corrected in the PDF and HTML versions of the paper.

