# Supplementary material for: Structural basis for the inhibitory effects of a novel reversible covalent ligand on PPARγ phosphorylation
Source: Sci Rep. 2019 Aug 1;9:11168. doi: 10.1038/s41598-019-47672-w (PMC6671948; doi:10.1038/s41598-019-47672-w)
Supplement: Supplementary file 1 — Supplementary information [file 41598_2019_47672_MOESM1_ESM.pdf]

<Supplementary Information>

Structural basis for the inhibitory effects of a novel reversible covalent ligand on PPARγ phosphorylation

Jun Young Jang[1], Hyunsoo Kim[2], Hyun-Jung Kim[3], Se Won Suh[2], Seung Bum Park[2], and Byung Woo Han[1]

[1]Research Institute of Pharmaceutical Sciences, College of Pharmacy, Seoul National University, Seoul 08826, Republic of Korea

[2]Department of Chemistry, College of Natural Sciences, Seoul National University, Seoul 08826, Republic of Korea

[3]College of Pharmacy, Chung-Ang University, Seoul 06974, Republic of Korea

Jun Young Jang and Hyunsoo Kim contributed equally.

Correspondence and requests for materials should be addressed to S.B.P. (sbpark@snu.ac.kr) or B.W.H. (bwhan@snu.ac.kr).

Contents

1. Supplementary Figures -- 2

2. Supplementary Tables -- 6

3. Supplementary Materials and Methods -- 8

1. Supplementary Figures

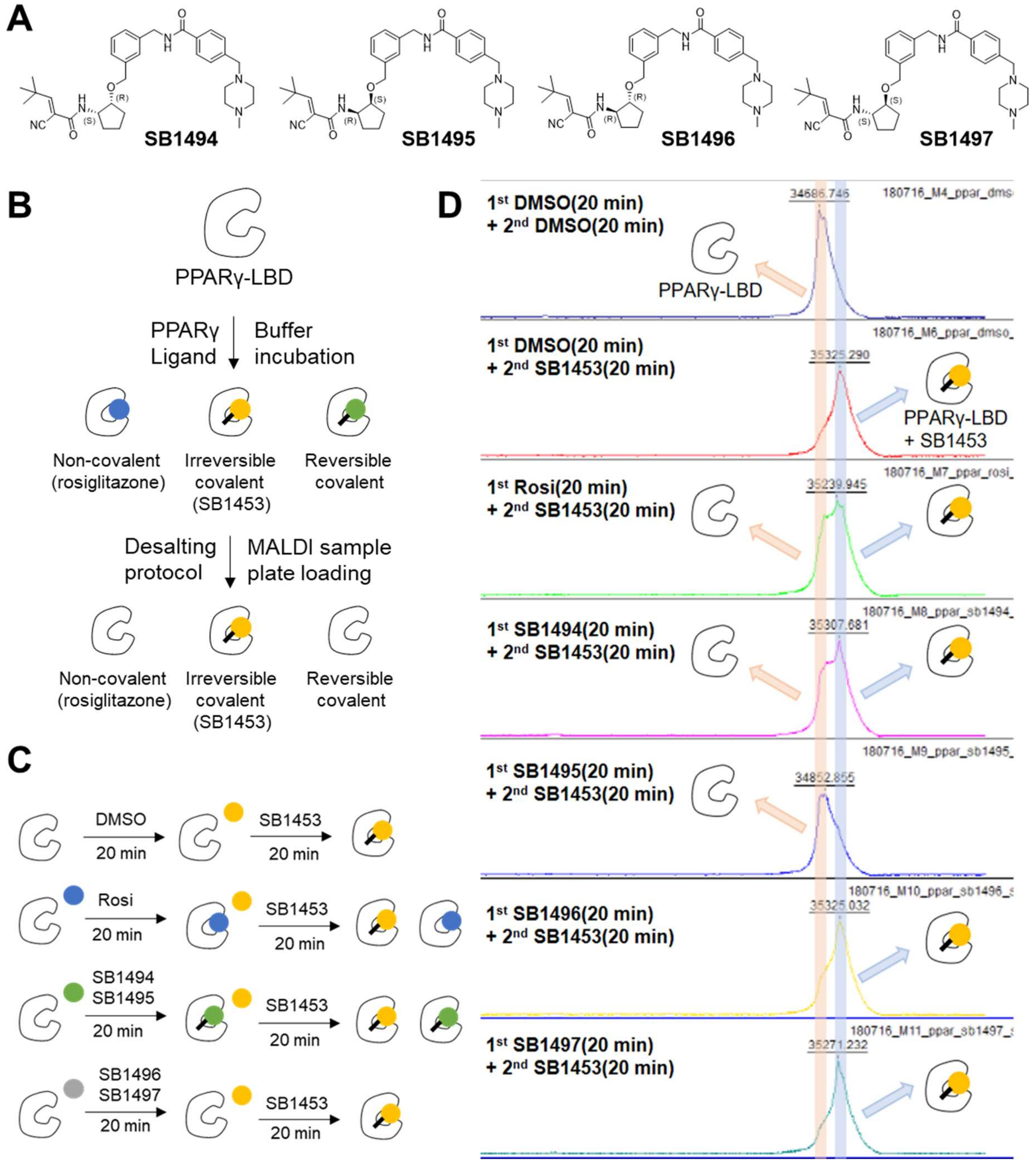

Supplementary Figure S1. (A) Chemical structures of SB1494, SB1495, SB1496, and SB1497. (B) General scheme of MALDI-TOF MS analysis of PPARγ-LBD and compound competitive binding experiment. (C) Schematic procedure of sequential ligand incubation test. (D) MALDI-TOF MS spectra of protein sample from PPARγ-LBD incubation test with the sequential treatment of indicated compounds.

We conducted a MALDI-TOF MS analysis to confirm whether four stereoisomers (SB1494–1497) do

bind with PPARγ-LBD or not. PPARγ-LBD (3.14 μM) was incubated with first compound (6.6 equiv.) in Tris-HCl (25 mM, pH 7.5) for 20 min. Then, each incubation tube was treated with SB1453 (13.2 equiv.) for 20 min at 30 °C. The resulting protein sample was analyzed by MALDI-TOF Mass spectrometer. As a result, we found that among four stereoisomers SB1494 and SB1495 showed the specific binding to PPARγ-LBD; SB1494 and SB1495 blocked the covalent binding of SB1453 with PPARγ via pre-occupation at the ligand binding pocket of PPARγ, while SB1496 and SB1497 were not able to interrupt the covalent binding event of SB1453.

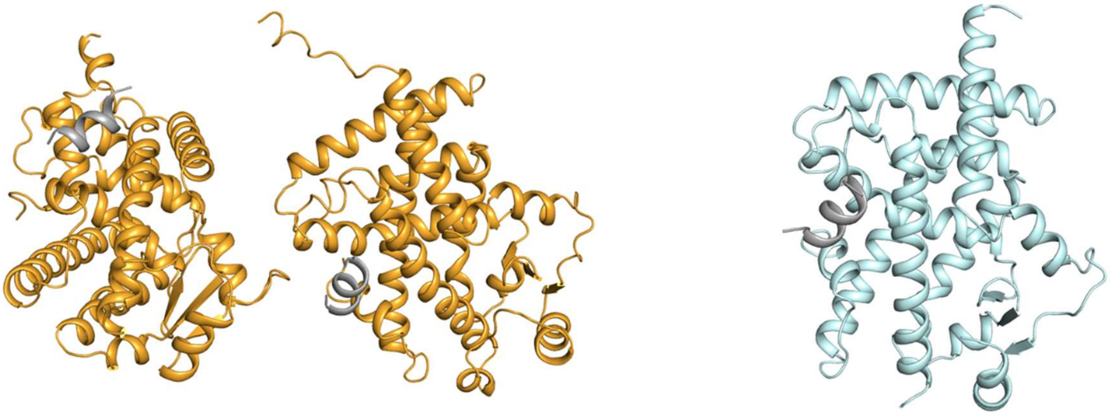

SB1495-bound PPARγ LBD structure in *orthorhombic* space group $P2_12_12_1$ (2 monomers in ASU)

SB1494-bound PPARγ LBD structure in *orthorhombic* space group $P2_12_12$ (1 monomer in ASU)

Supplementary Figure S2. SB1495- and SB1494-bound PPARγ LBD structures in the asymetric units.

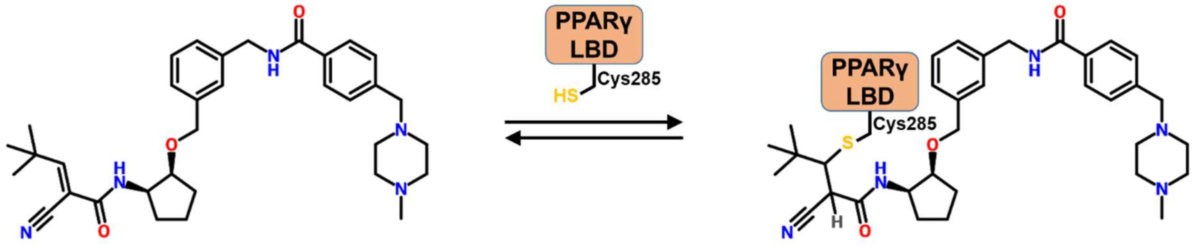

SB1495

Supplementary Figure S3. Mechanism of the reversible covalent addition reaction of Cys285 with the cyanoacrylamide moiety of SB1495.

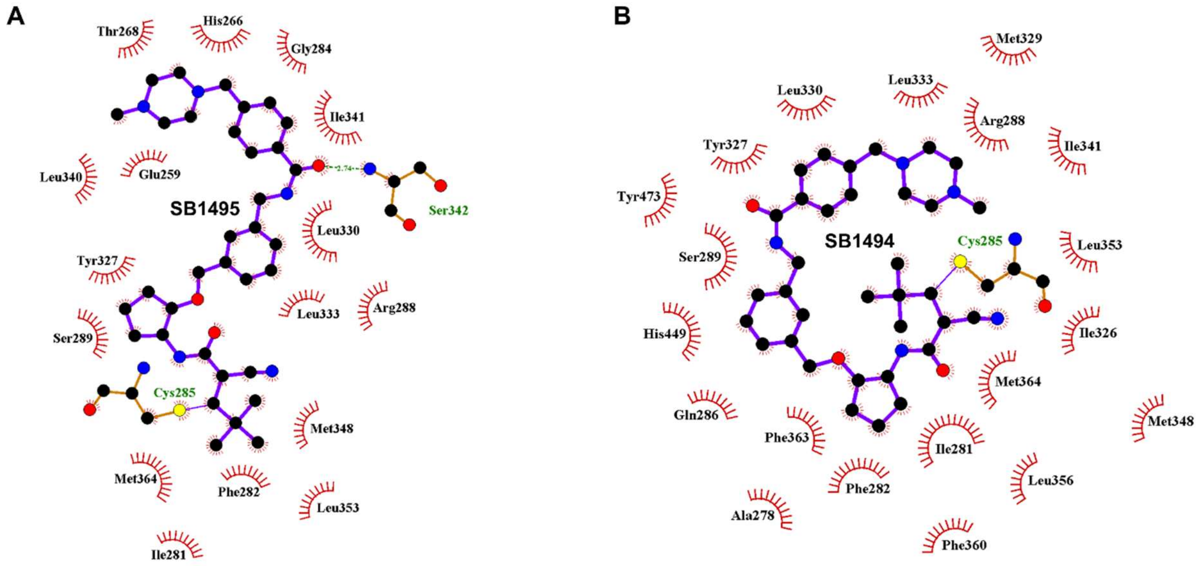

Supplementary Figure S4. Schematic representation of the interactions between PPARγ LBD and reversible covalent ligands SB1495 (A) and SB1494 (B), as calculated using LigPlot+ (Laskowski, R.A. and Swindells, M.B., *Journal of Chemical Information and Modeling*, (2011) 51:2778-2786). A hydrogen bond is depicted by dashed lines and labeled with the donor–acceptor distance in Å. Hydrophobic effects are indicated by spoked arcs and atoms with spokes. Oxygen, nitrogen, sulfur, and carbon atoms are colored in red, blue, yellow, and black, repectively.

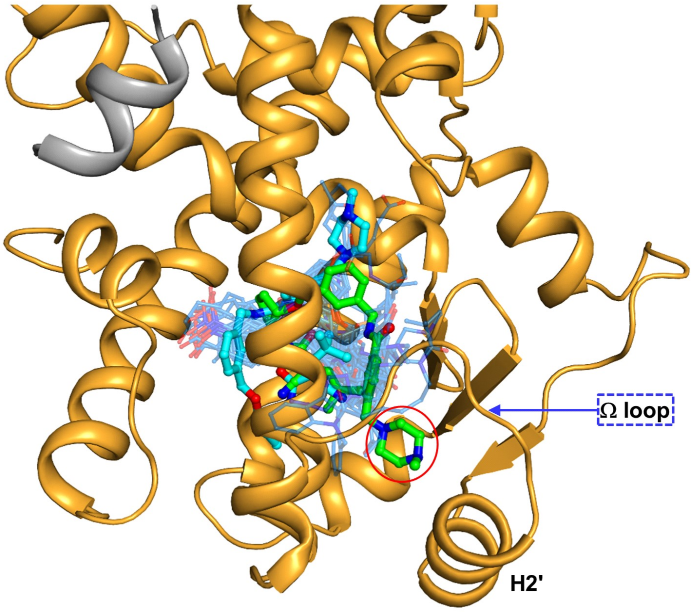

Supplementary Figure S5. Superposition of SB1495 and SB1494 with other covalent PPARγ ligands from known complex structures. A total of 22 covalent ligands to PPARγ LBD in PDB were superimposed onto the SB1495-bound structure (ribbon in bright orange). SB1495 and SB1494 are shown as green and cyan stick models, respectively, and the other covalent ligands are shown as marine lines. The piperazine moiety of SB1495 is highlighted in a red circle.

Unedited western blot images used in the main figure

Figure 1C.

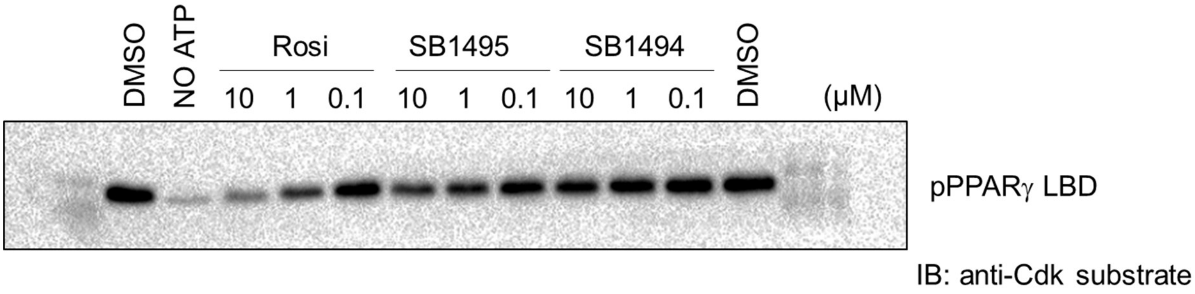

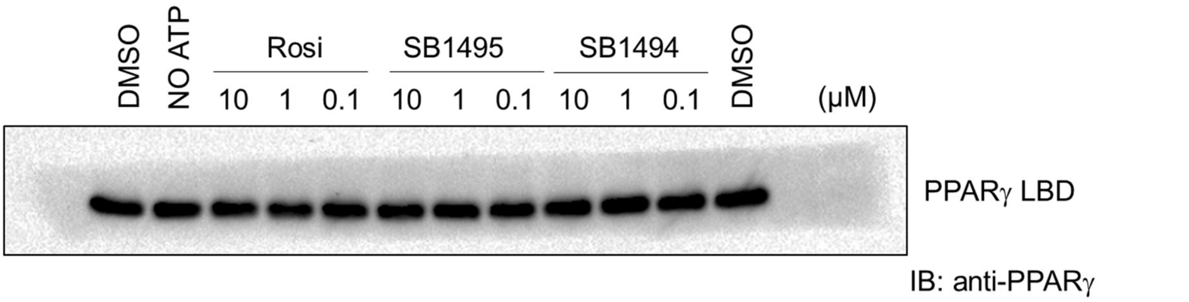

2. Supplementary Tables

Supplementary Table S1. Information of crystallization condition, space group, and unit cell parameters for the structures of PPARγ LBD mentioned in the manuscript.

PPARγ LBD structures	Crystallization condition	Space group	Unit cell parameters (Å)
SB1495-bound	60% (v/v) Tacsimate (pH 7.0)	$P2_12_12_1$	a = 62.4, b = 62.5, c = 162.2
Ligand-free	60% (v/v) Tacsimate (pH 7.0)	$P2_12_12$	a = 131.2, b = 53.4, c = 54.0
R35-bound (PDB ID: 5GTN)*	57.5% (v/v) Tacsimate (pH 7.0)	$P2_12_12$	a = 131.7, b = 52.5, c = 54.3
SB1494-bound	2.2 M sodium malonate (pH 7.0)	$P2_12_12$	a = 131.2, b = 53.3, c = 53.8
Agonist-free (PDB ID: 5GTP)*	2.2 M sodium malonate (pH 7.0)	$P2_12_12$	a = 131.3, b = 53.1, c = 53.6

* **Reference**: Jang, J. Y. *et al.* Structural basis for differential activities of enantiomeric PPAR gamma agonists: Binding of S35 to the alternate site. BBA-Proteins Proteom 1865, 674-681, doi:10.1016/j.bbapap.2017.03.008 (2017).

Supplementary Table S2. Statistics for the data collection and model refinement.

Model name	SB1495-bound (PDB ID: 6IJR)	SB1494-bound (PDB ID: 6IJS)	Ligand-free (PDB ID: 6JQ7)
A. Data collection			
X-ray source	PLS-7A	PLS-7A	PLS-11C
X-ray wavelength (Å)	0.97933	0.97933	0.97918
Space group	$P2_12_12_1$	$P2_12_12$	$P2_12_12$
Unit cell parameters			
a, b, c (Å)	62.36, 62.45, 162.16	131.20, 53.32, 53.84	131.15, 53.41, 54.01
$\alpha = \beta = \gamma$ (°)	90	90	90
Resolution range (Å)	50.0 - 2.85 (2.90 - 2.85)	50.0 - 2.15 (2.19 - 2.15)	50.0 - 2.55 (2.59 - 2.55)
Total / unique reflections	83,470 / 15,503	209,189 / 21,339	116,850 / 13,063
Completeness (%)	99.5 (100.0)	99.9 (100.0)	99.6 (99.8)
$\langle I / \sigma_I \rangle$	28.7 (3.0)	41.0 (3.6)	18.3 (3.0)
R_{merge} (%)	8.7 (78.3)	8.7 (72.1)	10.2 (76.2)
$CC_{1/2}$	0.962 (0.828)	0.971 (0.854)	1.000 (0.830)
B. Model refinement			
Resolution range (Å)	50.0–2.85	30.0–2.15	30.0–2.55
R_{work} / R_{free} (%)	23.6 / 26.6	20.4 / 24.1	22.0 / 25.7
No. of non-hydrogen atoms			
Protein	4691	2208	2185
Ligand	84	42	-
Water oxygen	24	82	48
Average *B* factor (Å^2)			
Protein	90.0	55.5	40.7
Ligand	83.2	67.3	-
Water oxygen	53.4	56.2	28.7
R.m.s. deviations from ideal geometry			
Bond lengths (Å)	0.009	0.009	0.009
Bond angles (°)	1.47	1.48	1.34
Ramachandran plot			
Favored / Outliers (%)	97.0 / 0.0	97.4 / 0.0	97.0 / 0.0
Poor rotamers (%)	0.00	0.00	0.00
Values in parentheses refer to the highest resolution shell.			

3. Supplementary Materials and Methods

All methods were performed in accordance with the relevant guidelines and regulations.

Reagents. Rosiglitazone (R0106, TCI) was purchased from commercial venders and used without further purification.

MALDI-TOF MS analysis. All MALDI-TOF experiments were performed by using an ultrafleXtreme [Bruker, Germany]. Purified PPARγ-LBD protein was diluted in assay buffer (25 mM Tris-HCl, pH 7.5, 5 mM β-glycerophosphate, 2 mM DTT, 0.1 mM Na_3VO_4, 10 mM $MgCl_2$) and compounds were treated to PPARγ-LBD for 20 min at 30 °C. The protein sample was prepared by using Millipore $ZipTip_{C18}$ according to manufacturer's procedure and mixed with matrix solution (alpha-Cyano-4-hydroxycinnamic acid in 50% acetonitrile and 0.1% trifluoroacetic acid). The mixture sample was spotted on the MALDI target plate, and the spectrum was acquired in positive ion mode.
